# Orally administered gold nanoparticles protect against colitis by attenuating Toll-like receptor 4- and reactive oxygen/nitrogen species-mediated inflammatory responses but could induce gut dysbiosis in mice

**DOI:** 10.1186/s12951-018-0415-5

**Published:** 2018-11-01

**Authors:** Suqin Zhu, Xiumei Jiang, Mary D. Boudreau, Guangxin Feng, Yu Miao, Shiyuan Dong, Haohao Wu, Mingyong Zeng, Jun-Jie Yin

**Affiliations:** 1 College of Food Science and Engineering, Ocean University of China, 5 Yushan Road, Qingdao, 266003 Shandong China; 2 Division of Analytical Chemistry, Office of Regulatory Science, Center for Food Safety and Applied Nutrition, U.S. Food and Drug Administration, College Park, MD 20740 USA; 3 Division of Biochemical Toxicology, National Center for Toxicological Research, U.S. Food and Drug Administration, Jefferson, 72079 AR USA; 4 Department of Clinical Laboratory, The Affiliated Hospital of Qingdao University, Qingdao, 266003 Shandong China

**Keywords:** Gold nanoparticles, Inflammatory bowel disease, Gut microbiota, Reactive oxygen/nitrogen species, Toll-like receptors

## Abstract

**Background:**

Gold nanoparticles (AuNPs) are attracting interest as potential therapeutic agents to treat inflammatory diseases, but their anti-inflammatory mechanism of action is not clear yet. In addition, the effect of orally administered AuNPs on gut microbiota has been overlooked so far. Here, we evaluated the therapeutic and gut microbiota-modulating effects, as well as the anti-inflammatory paradigm, of AuNPs with three different coatings and five difference sizes in experimental mouse colitis and RAW264.7 macrophages.

**Results:**

Citrate- and polyvinylpyrrolidone (PVP)-stabilized 5-nm AuNPs (Au-5 nm/Citrate and Au-5 nm/PVP) and tannic acid (TA)-stabilized 5-, 10-, 15-, 30- and 60-nm AuNPs were intragastrically administered to C57BL/6 mice daily for 8 days during and after 5-day dextran sodium sulfate exposure. Clinical signs and colon histopathology revealed more marked anti-colitis effects by oral administration of Au-5 nm/Citrate and Au-5 nm/PVP, when compared to TA-stabilized AuNPs. Based on colonic myeloperoxidase activity, colonic and peripheral levels of interleukin-6 and tumor necrosis factor-α, and peripheral counts of leukocyte and lymphocyte, Au-5 nm/Citrate and Au-5 nm/PVP attenuated colonic and systemic inflammation more effectively than TA-stabilized AuNPs. High-throughput sequencing of fecal 16S rRNA indicated that AuNPs could induce gut dysbiosis in mice by decreasing the α-diversity, the Firmicutes/Bacteroidetes ratio, certain short-chain fatty acid-producing bacteria and *Lactobacillus*. Based on in vitro studies using RAW264.7 cells and electron spin resonance oximetry, AuNPs inhibited lipopolysaccharide (LPS)-triggered inducible nitric oxide (NO) synthase expression and NO production via reduction of Toll-like receptor 4 (TLR4), and attenuated LPS-induced nuclear factor kappa beta activation and proinflammatory cytokine production via both TLR4 reduction and catalytic detoxification of peroxynitrite and hydrogen peroxide.

**Conclusions:**

AuNPs have promising potential as anti-inflammatory agents; however, their therapeutic applications via the oral route may have a negative impact on the gut microbiota.
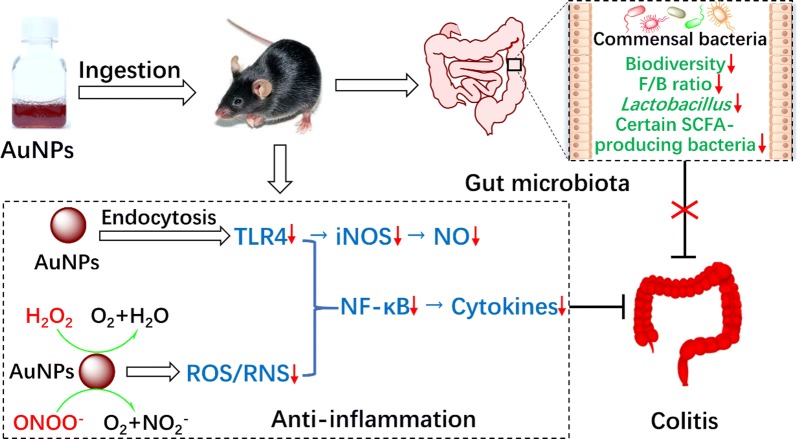

**Electronic supplementary material:**

The online version of this article (10.1186/s12951-018-0415-5) contains supplementary material, which is available to authorized users.

## Introduction

Gold is well known for its use in jewelry, utensils, and monetary currency, but its therapeutic applications in traditional medicines date back to 2500 BC [1]. Since the 1890s, gold has attracted a lot of attention in modern medicine as a therapeutic agent to treat many autoimmune, infectious, and neoplastic diseases [2]. Gold(I) compounds like sodium aurothiomalate (Myocrysin), aurothioglucose (Solganol), and auranofin (Ridaura) are the most commonly used gold-based drugs; nevertheless, their adverse side effects (e.g. skin rashes, leukopenia, thrombocytopenia and anemia) affect as many as 40% of patients [3]. Fortunately, nanotechnology offers a safer and more effective alternative, i.e. colloidal gold. When administered orally, subcutaneously, or intra-articularly, gold nanoparticles (AuNPs) were shown to be more effective and less toxic than conventional gold(I) drugs in the treatment of rheumatoid arthritis in human and animal studies [4–6]. Therefore, exploration of AuNPs in chrysotherapy is attracting the attention of researchers in the field.

Inflammatory bowel disease (IBD), including ulcerative colitis and Crohn’s disease, affects up to 0.5% of the western population and is becoming increasingly prevalent in newly industrialized countries [7]. IBD is a relapsing and incurable disease that is featured by chronic intestinal inflammation and displays a variety of symptoms, such as persistent diarrhea, abdominal pain, and fatigue. AuNPs have been shown to play an anti-inflammatory role in mouse obesity, rat arthritis, and rat focal cerebral ischemia–reperfusion [8–11], and AuNPs can inhibit innate immune activation in macrophages in vitro by attenuating nuclear factor kappa beta (NF-κB), interferon-β/signal transducer and activator of transcription 1 (STAT1), and Toll-like receptor 9 signaling pathways [12–14]. The oral administration of AuNPs exerts little toxicity [15, 16] and, with the gastrointestinal tract as their initial point of contact, AuNPs may have the potential to attenuate IBD.

The present study evaluated the anti-inflammatory potential of citrate-, polyvinylpyrrolidone (PVP)-, and tannic acid (TA)-stabilized AuNPs (5 to 60 nm diameter) in vivo, when administered by oral gavage to a C57BL/6 mouse model of dextran sodium sulphate (DSS)-induced colitis, and in vitro, when incubated with the murine macrophage cell line, RAW264.7. The effect of oral administration of AuNPs on mouse gut microbiota was also examined in the in vivo study.

## Results and discussion

### Particle characterization

The core sizes of AuNPs were evaluated by transmission electron microscopy (TEM) (Additional file 1: Figure S2), and were found to correspond well with those declared by the manufacturers. The hydrodynamic size and zeta potential of AuNPs dispersed in water and 10% fetal bovine serum (FBS)-supplemented Dulbecco’s Modified Eagle medium (DMEM) are summarized in Table 1. PVP-stabilized 5-nm AuNPs (Au-5 nm/PVP) showed a near zero zeta potential in water, confirming that PVP is a neutral coating that confers colloidal stability by steric repulsion. Citrate and TA are anionic coatings that prevent particle aggregation via electrosteric stabilization, and this was confirmed by the strongly negative zeta potentials of citrate- and TA-coated AuNPs in water (Table 1).

**Table 1:** Particle size and zeta potential of gold nanoparticles (AuNPs)

AuNPs	Size by DLS^a^ Intensity-weighted peak (nm)		Zeta potential^b^	
	Water	DMEM + FBS	Water	DMEM + FBS
Au-5 nm/PVP	8.7 ± 0.4	92.1 ± 1.4	0.2 ± 0.7	0.2 ± 1.1
Au-5 nm/Citrate	8.6 ± 0.6	173.2 ± 2.4	− 30.8 ± 2.7	− 3.5 ± 0.7
Au-5 nm/TA	11.6 ± 0.9	90.0 ± 1.5	− 37.4 ± 9.6	− 6.0 ± 0.4
Au-10 nm/TA	18.3 ± 1.7	88.4 ± 1.4	− 36.7 ± 5.1	− 5.8 ± 0.8
Au-15 nm/TA	25.6 ± 1.2	58.8 ± 1.2	− 23.3 ± 5.7	− 6.2 ± 0.6
Au-30 nm/TA	56.5 ± 1.4	78.1 ± 1.1	− 25.3 ± 1.7	− 5.8 ± 0.5
Au-60 nm/TA	89.8 ± 2.4	108.5 ± 2.5	− 25.0 ± 0.3	− 6.1 ± 0.5

Data are presented as the mean ± standard deviation of three replicates

*DLS* dynamic light scattering, *DMEM* Dulbecco’s modified Eagle medium, *FBS* fetal bovine serum

^a^The DLS measurement was performed at 1/40 OD of AuNPs

^b^The zeta potential was measured at 1/10 OD of AuNPs

Once in contact with gastrointestinal juices, blood, lymph, or any other biological liquid, nanoparticles encounter changes in surface chemistry owing to the formation of a protein “corona” and alterations in pH and salinity. When compared to water, the zeta potentials for TA- and citrate-coated AuNPs were greatly reduced when evaluated in 10% FBS-supplemented DMEM, suggesting a charge shielding effect by serum adsorption. In complete media, citrate-stabilized 5-nm AuNPs (Au-5 nm/Citrate) yielded a hydrodynamic size as high as 173.2 nm and was much higher than those of Au-5 nm/PVP (92.1 nm) and TA-stabilized 5-nm AuNPs (Au-5 nm/TA) (90.0 nm), suggesting that citrate-coated AuNPs showed a greater extent of aggregation under physiological salt conditions than AuNPs stabilized with PVP or TA. Interestingly, the larger TA-coated AuNPs seemed to aggregate to a lesser extent under physiological salt conditions than in water, based on the observed hydrodynamic size shifts in the complete media.

A bacterial endotoxin test is usually required before preclinical in vitro and in vivo studies of pharmaceutical formulations. The endotoxin levels in the commercial preparations of AuNPs were below the limit of detection (< 0.005 EU/mL).

### Effects of orally administered AuNPs on DSS-induced colitis

**Fig. 1:**
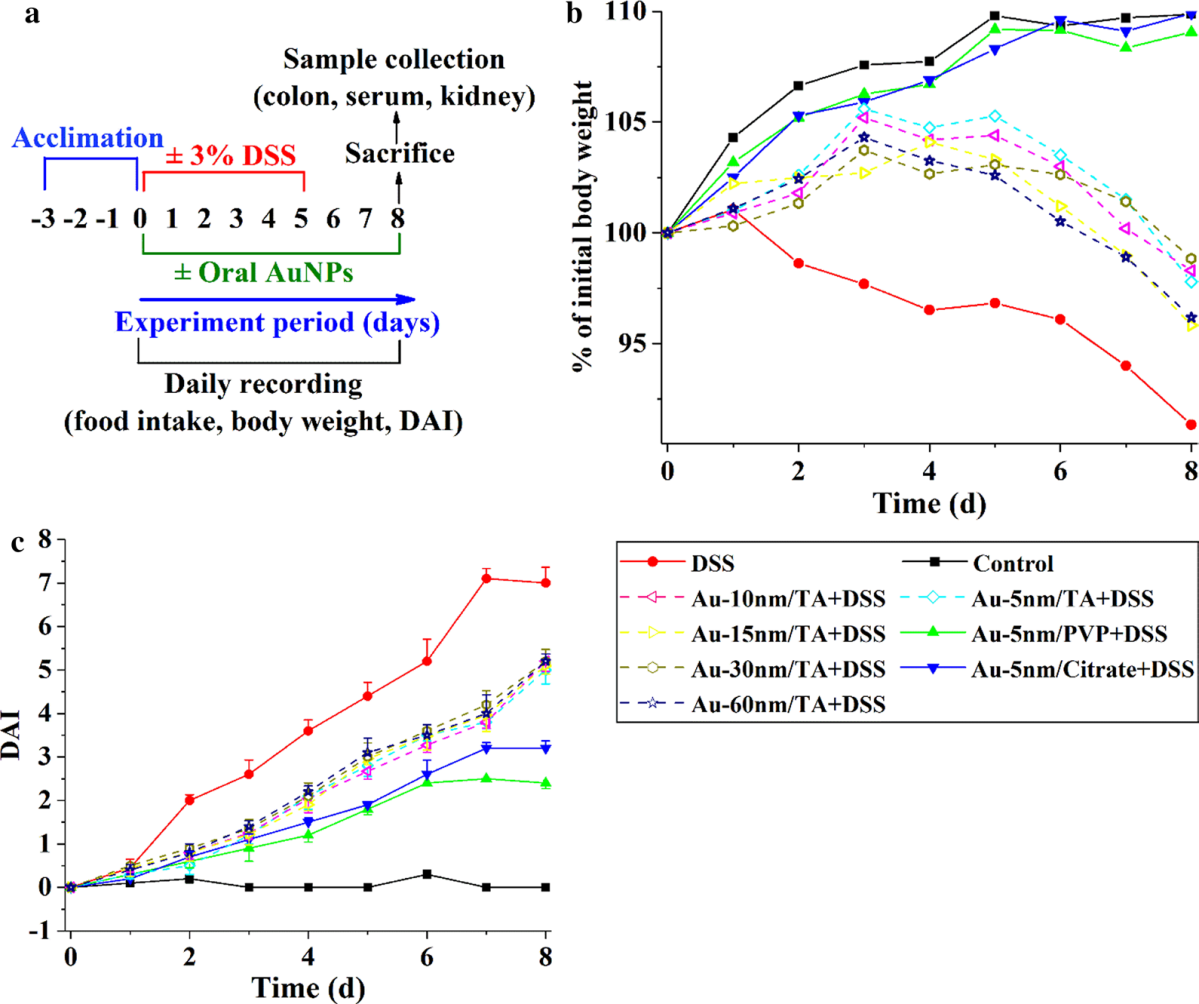
Clinical parameters of DSS-induced colitis: **a** the experimental plan; **b** body weight changes, represented as means of each group with standard deviations of < 6.6; **c** Disease Activity Index (DAI), represented as means ± standard deviations

To assess their potential therapeutic efficacy on colitis, AuNPs were administered by oral gavage to the DSS-treated mice for 8 days (Fig. 1a). To ensure a fair comparison of the activities of different sized AuNPs, we dosed mice with nanoparticles at equivalent optical densities (OD) of their surface plasmon resonance (SPR) peaks so that the gold colloids possessed essentially equivalent effective surface areas.

The kidney is a major site for organ accumulation of orally administrated AuNPs following their intestinal absorption [17–19]. According to Choi et al., nanoparticles should have final hydrodynamic diameters ≤ 5.5 nm to be filtered from the kidney [20]. In this study, according to their hydrodynamic sizes in DMEM medium supplemented with 10% FBS (Table 1), AuNPs were too large to be excreted from the mouse body by the renal route, so the accumulation of gold was determined in the kidneys of mice to indirectly measure the bioavailability of orally administrated AuNPs (Table 2). The kidneys of mice in the Au-5 nm/PVP group had significantly greater accumulations of gold than did the kidneys of mice in the Au-5 nm/Citrate and Au-5 nm/TA groups (least significant difference (LSD), *P* < 0.05). A size-dependent accumulation of gold was observed for TA-coated AuNPs, with peak accumulation observed for TA-stabilized 15-nm AuNPs (Au-15 nm/TA) (LSD, *P* < 0.05). These results suggest a coating- and size-dependent bioavailability for orally administered AuNPs.

**Table 2:** The accumulation of gold in the kidney after oral administration of AuNPs in mice

AuNPs	Approximate doses (μg/kg body weight/day)	Au contents in kidney (pg/g wet weight)^1^
Au-5 nm/PVP	25	158.6 ± 27.9^c^
Au-5 nm/Citrate	25	128.9 ± 15.6^d^
Au-5 nm/TA	25	119.5 ± 19.1^d^
Au-10 nm/TA	22	143.5 ± 16.9^cd^
Au-15 nm/TA	20	265.2 ± 28.6^a^
Au-30 nm/TA	18	239.3 ± 19.8^b^
Au-60 nm/TA	15	121.3 ± 28.3^d^

^1^Values are means ± standard deviations. Means in a column without a common superscript letter (a, b, c and d) are statistically different (ANOVA followed by LSD test, *P* < 0.05)

The DSS control group had significantly lower mean total food intake (29.1 ± 1.3 g/mouse) during the entire experiment than the normal control group (36.2 ± 2.5 g/mouse) (Dunnett’s, *P* < 0.05), while the mean total food intakes (g/mouse) of mice in the AuNPs groups were similar to the normal controls (data not shown).

Figure 1b shows the kinetics of body weight during the entire experiment. The DSS control group suffered from significant weight loss compared to the normal control group from day 2 (Dunnett’s, *P* < 0.01). The AuNP-treated animals had significantly less weight loss than the DSS control-treated ones from day 3 (Dunnett’s, *P* < 0.01). Au-5 nm/PVP and Au-5 nm/Citrate displayed superior protecting activities than TA-coated AuNPs from day 6 (LSD, *P* < 0.01). No significant size dependence in body weight was observed for TA-coated AuNPs.

The Disease Activity Index (DAI) values were obtained based on the pathological conditions of weight loss, loose stool and fecal blood (Fig. 1c). The DSS treatment caused a rise in DAI from day 2 (Dunnett’s, *P* < 0.01), and this was ameliorated by oral administration of PVP- and citrate-coated 5-nm AuNPs from day 3 (Dunnett’s, *P* < 0.01) and TA-coated AuNPs from day 6 (Dunnett’s, *P* < 0.01). Au-5 nm/PVP and Au-5 nm/Citrate displayed remarkably better protecting effects than TA-coated AuNPs from day 4 (LSD, *P* < 0.01). TA-coated AuNPs showed no significant size dependence in DAI.

**Fig. 2:**
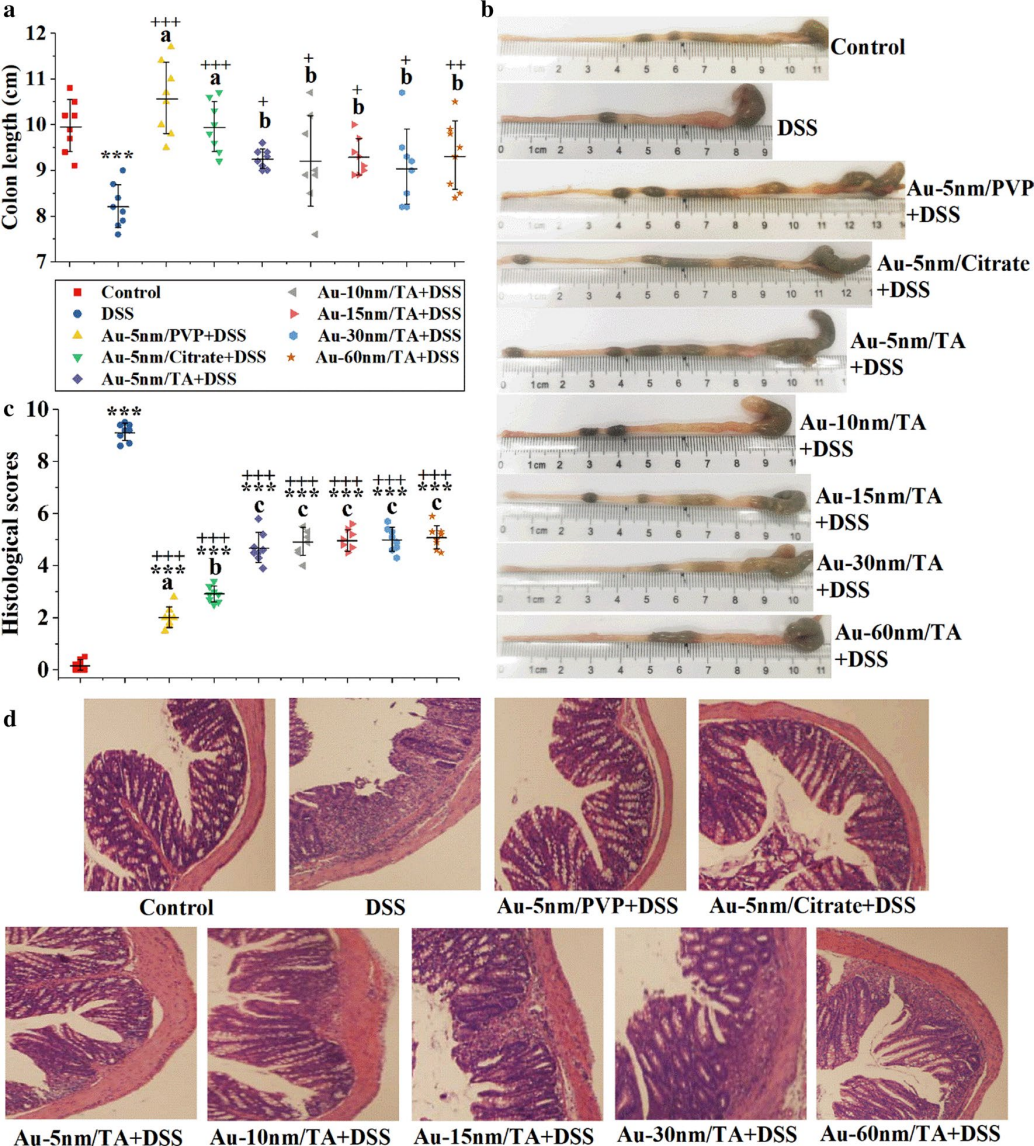
Histological observation of the colon: **a** colon length; **b** representative images of the measurements of colon length; **c** histological scores and **d** typical images of H&E stained colonic sections. Each point in **a** and **c** represents a single sample. Asterisks and plus signs represent significant differences (***P < 0.001, ^+++^*P* < 0.001, ^++^*P* < 0.01, ^+^*P* < 0.05, Dunnett’s post hoc test following ANOVA) versus the normal control and the DSS control, respectively. Different lower-case letters mark significant differences (*P* < 0.05, ANOVA followed by LSD test) between AuNP-treated groups

Figure 2a, b show that the oral administration of AuNPs improved the DSS-induced colon shortening (Dunnett’s, *P* < 0.05), with Au-5 nm/PVP and Au-5 nm/Citrate showing higher amending effects than TA-coated AuNPs (LSD, *P* < 0.05). Histological analyses of hematoxylin and eosin (H&E) stained colon tissue (Fig. 2d) revealed that the oral administration of AuNPs ameliorated the DSS-induced thinned and disordered mucosal structure and decreased the area of inflammatory cell infiltration. By using histological scoring, we found that Au-5 nm/PVP and Au-5 nm/Citrate were more efficient in attenuating DSS-induced histological lesions than TA-coated AuNPs (LSD, *P* < 0.05) (Fig. 2c). There was no significant size dependence in colon length and histological score for TA-coated AuNPs.

AuNPs, apparently, exerted their anti-colitis activities in a coating-rather than size-dependent manner, based on the results observed for body weight changes, DAI, and colon histology. Importantly, TA-coated AuNPs had less anti-colitis potential than PVP- and citrate-coated ones. AuNPs were dosed at concentrations of 1/25 OD, i.e. 2.8 mg gold/L or less, which were neglectable compared to that of DSS (30 g/L) in drinking water, so they were unlikely to interfere with DSS to prevent colitis induction. Anti-inflammation and gut microbiota modulation are typical anti-colitis mechanisms, and they will be examined in the following parts to elucidate the mechanism of action of AuNPs.

### Oral administration of AuNPs reduced colonic and systemic inflammation

**Fig. 3:**
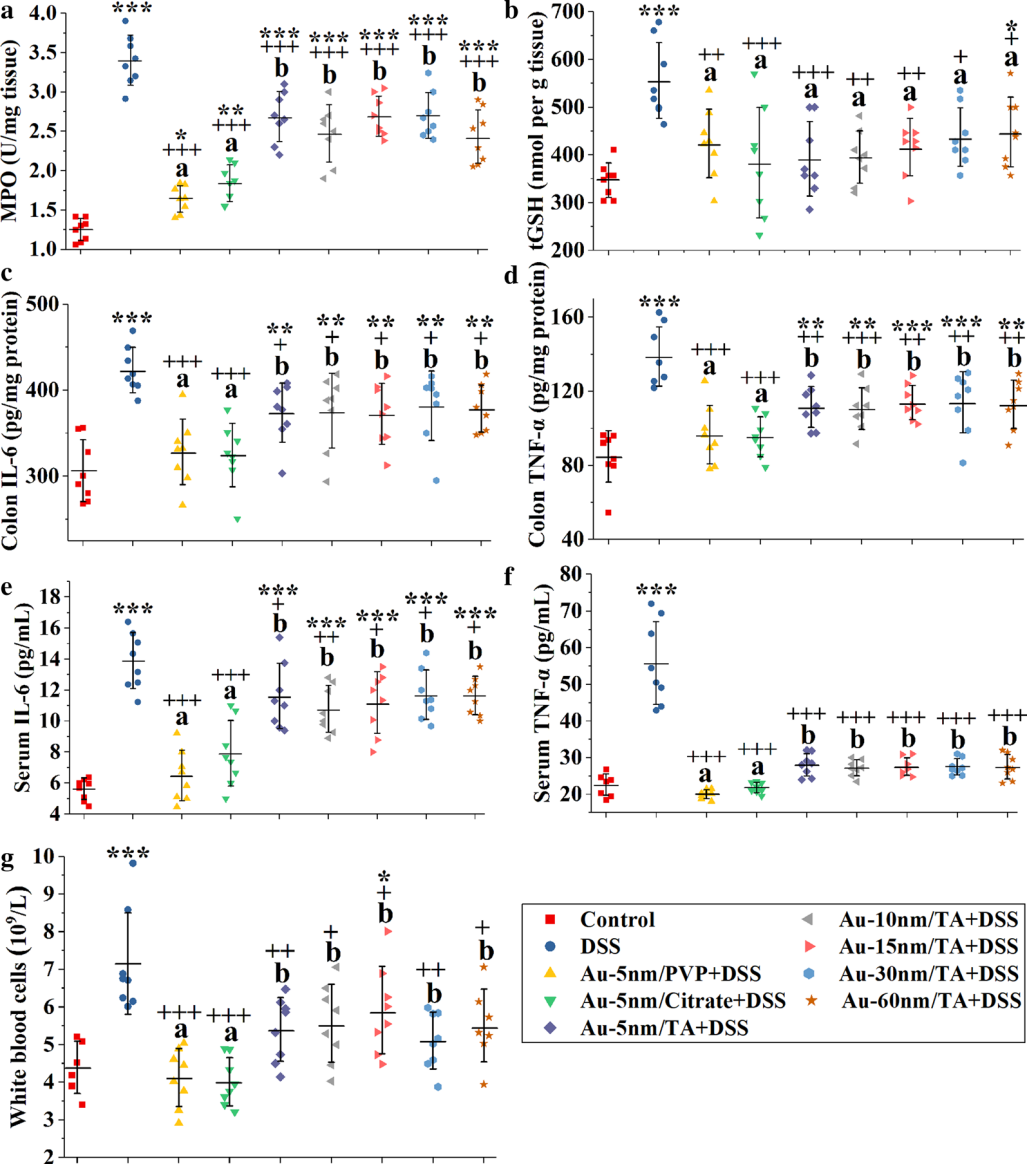
Biochemical and hematological parameters of DSS-induced colitis: the colonic levels of **a** MPO activity, **b** tGSH, **c** IL-6 and **d** TNF-α; the serum levels of **e** IL-6 and **f** TNF-α; the hematologic results of (g) white blood cells. Each point represents a single sample. Asterisks and plus signs represent significant differences (***P < 0.001, **P < 0.01, *P < 0.05, ^+++^*P* < 0.001, ^++^*P* < 0.01, ^+^*P* < 0.05, Dunnett’s post hoc test following ANOVA) versus the normal control and the DSS control, respectively. Different lower-case letters mark significant differences (*P* < 0.05, ANOVA followed by LSD test) between AuNP-treated groups

Myeloperoxidase (MPO) is present in large quantities in neutrophils, and its activity is a widely used parameter for evaluating inflammatory cell infiltration [21]. As shown in Fig. 3a, compared to the normal control group, the DSS control group had significantly increased colonic MPO activity (Dunnett’s, *P* < 0.001). In contrast, the oral administration of AuNPs (Dunnett’s, *P* < 0.001) suppressed much of the MPO activity induced by DSS, with Au-5 nm/PVP and Au-5 nm/Citrate showing enhanced inhibitory effects compared to TA-coated AuNPs (LSD, *P* < 0.05).

The rate of glutathione consumption may increase dramatically in many chronic inflammatory diseases and is associated with the role of glutathione in protecting cells against oxidative injuries [22]. DSS induced an increase in colonic total glutathione (tGSH, reduced plus oxidized forms) levels (Dunnett’s, *P* < 0.001), and this increase was significantly inhibited by the oral administration of AuNPs (Dunnett’s, *P* < 0.05) (Fig. 3b).

Interleukin-6 (IL-6) and tumor necrosis factor-α (TNF-α) are important mediators of colonic and systemic inflammation in patients with IBD [23]. As shown in Fig. 3c–f, the oral administration of AuNPs was effective in ameliorating the increased production of IL-6 and TNF-α by DSS in both colon and serum (Dunnett’s, *P* < 0.05). Au-5 nm/PVP and Au-5 nm/Citrate were more efficient at attenuating colonic and serum levels of IL-6 and TNF-α than the TA-coated AuNPs (LSD, *P* < 0.05).

Elevated levels of white blood cells are usually associated with an inflammatory response. The number of white blood cells was elevated in the DSS control group (Dunnett’s, *P* < 0.01) (Fig. 3g), suggesting that significant systemic inflammation was caused by DSS treatment. The increased number of white blood cells induced by DSS was ameliorated by the oral administration of AuNPs (Dunnett’s, *P* < 0.05). Au-5 nm/PVP and Au-5 nm/Citrate were more effective than the TA-coated AuNPs at suppressing the increased number of white blood cells (LSD, *P* < 0.05).

The results of biochemical and hematological parameters revealed a coating- rather than size-dependent anti-inflammatory effect of AuNPs, with TA-coated AuNPs displaying weaker anti-inflammatory efficacy than PVP- and citrate-coated AuNPs. These findings aligned with the results of the anti-colitis activities and demonstrate that AuNPs attenuate DSS-induced colitis at least in part via anti-inflammatory activities.

### AuNPs altered gut microbiota profile in DSS-induced colitis mice

The gut microbiota has been shown to play an important role in the pathogenesis of IBD [24]. To investigate the effect of oral administration of AuNPs on mouse gut microbiota, high-throughput sequencing was employed to analyze the bacterial diversity in fecal samples from the groups of normal control, DSS control and Au-5 nm/TA. In total, 6327 Operational Taxonomic Units (OTUs) were obtained from 1,175,085 sequences of the fecal samples. A rarefaction analysis was performed to ascertain a complete recovery of the bacterial families in these samples (Additional file 1: Figure S3), and the rarefaction curves reached a plateau, suggesting that most of the bacteria taxa present in the fecal samples were recovered.

**Fig. 4:**
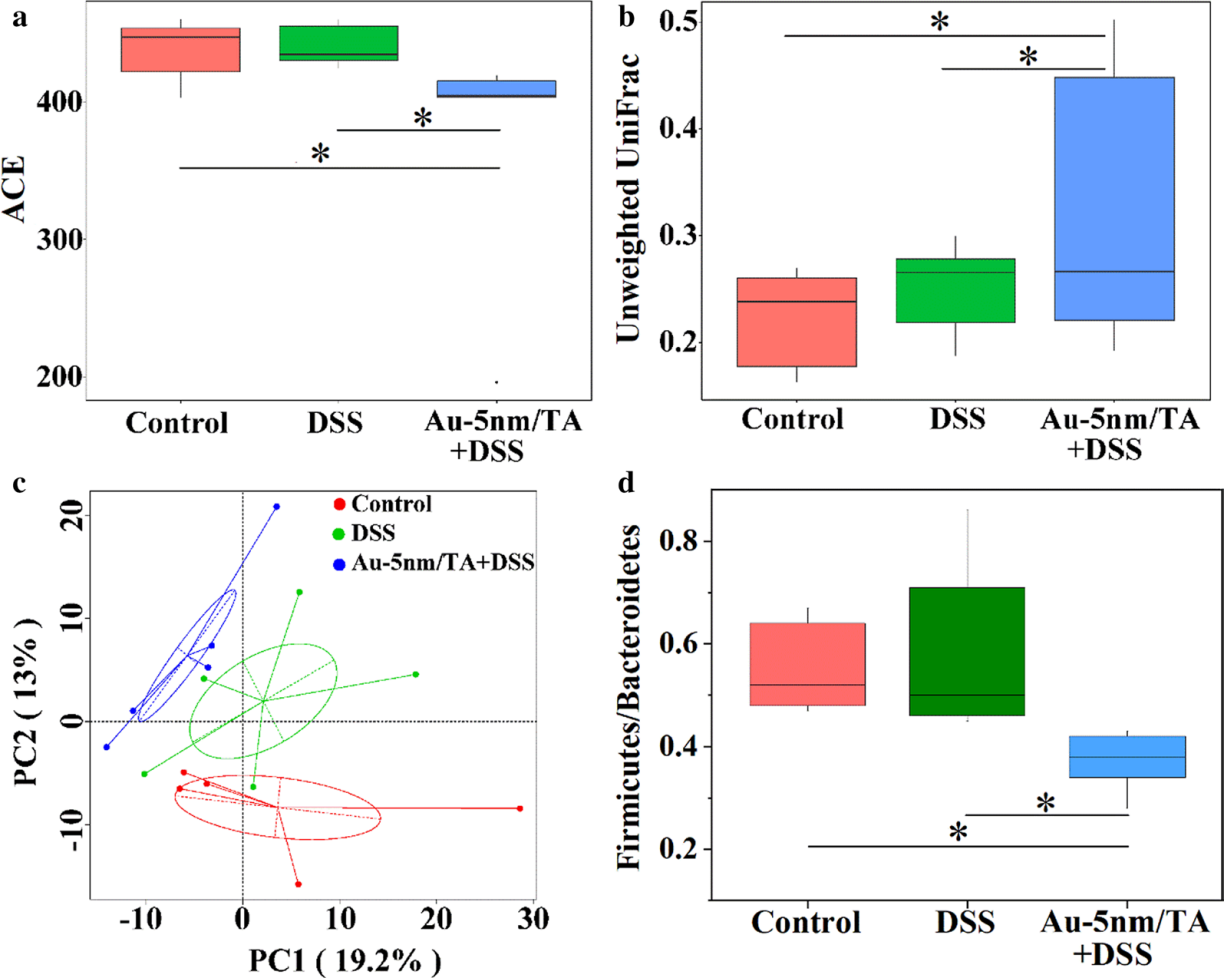
The effects of orally administered AuNPs on global gut microbiota structures: **a** the α-diversity metric ACE; **b** the unweighted UniFrac metric; **c** PCA analysis of β-diversity based upon the unweighted UniFrac distances, where each point represents a single sample with the percentages indicating the contribution of principal components to the difference of samples; **d** the ratios of Firmicutes to Bacteroidetes. The comparison is made by Wilcox’s test, and significant P-value is marked as *P < 0.05

The α-diversity (i.e. phylotype richness within each sample) was measured by abundance-based coverage estimator (ACE) (Fig. 4a). There was no significant difference in α-diversity between the normal control group and the DSS control group. The Au-5 nm/TA group displayed a significantly lower α-diversity than the normal control group (Wilcox, *P *< 0.05) and the DSS control group (Wilcox, *P *< 0.05), suggesting that oral administration of AuNPs markedly decreased the species richness of gut microbiota in mice. In fact, many studies consistently report a reduced biodiversity in the fecal microbiome of patients with IBD [25], while the oral administration of Au-5 nm/TA provided no benefit to the preservation of gut microbiota diversity.

The unweighted unique fraction (UniFrac) metric, which measures the phylogenetic distance (ranging from 0 to 1) between sets of taxa in a phylogenetic tree [26], was used to estimate the intragroup similarity of gut microbiota profile (Fig. 4b). No significant difference in this metric was observed between the groups of normal control and DSS control, suggesting an equivalent level of community structural similarity in these groups. The Au-5 nm/TA group presented greater unweighted UniFrac distances than the normal control group (Wilcox, *P *< 0.05) and the DSS control group (Wilcox, *P *< 0.05), indicating that oral administration of AuNPs increased the compositional dissimilarity of gut microbiota in mice.

The β-diversity (between sample diversity) was evaluated by a principal component analysis (PCA) analysis based upon unweighted UniFrac distances (Fig. 4c). The three groups clustered separately, suggesting different community structures of gut microbiota in these groups, although the Au-5 nm/TA group seemed to have a closer phylogenetic relationship with the DSS control group than with the normal control group. Firmicutes and Bacteroidetes are the two most abundant bacterial phyla in human and mouse fecal microbiota, and decreased Firmicutes or increased Bacteroidetes has been associated with aggravated intestinal inflammation in IBD [24]. The ratios of Firmicutes to Bacteroidetes (F/B) in the Au-5 nm/TA group were significantly lower than those in the DSS control group (Wilcox, *P* < 0.05) and the normal control group (Wilcox, *P* < 0.05) (Fig. 4d), suggesting the potential for a negative impact on gut bacteria by the oral administration of AuNPs.

**Fig. 5:**
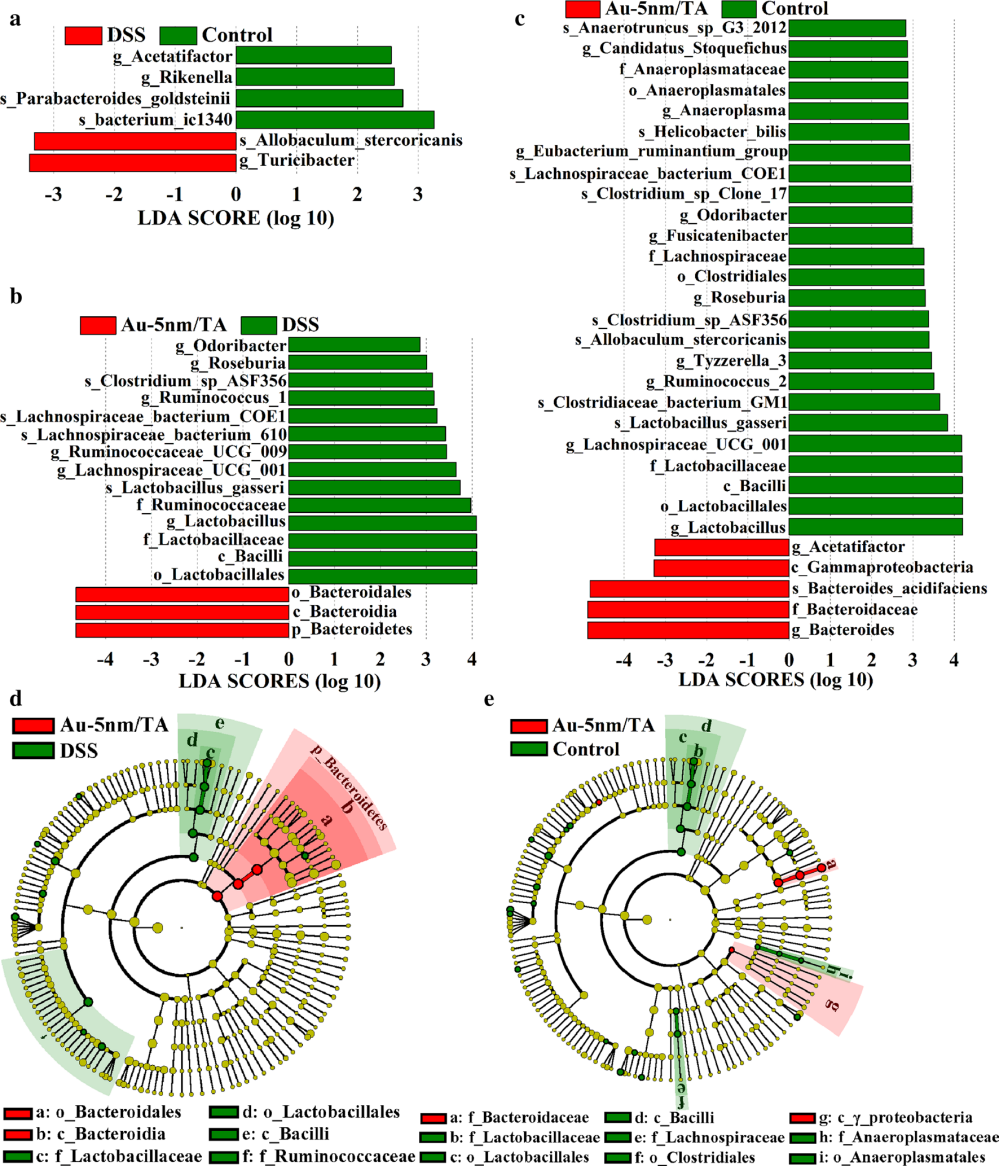
Key phylotypes of gut microbiota responding to the oral administration of Au-5 nm/TA as identified using LEfSe: **a**–**c** LDA scores of the differentially abundant taxa; **d**, **e** the cladograms based on LEfSe analysis with red and green circles meaning differences in relative abundance and yellow circles meaning non-significant differences

The linear discriminant analysis (LDA) effect size (LEfSe) algorithm unveiled 6, 17, and 30 significantly different taxa at different taxonomic levels between the groups of normal control and DSS control, Au-5 nm/TA and DSS control, and normal control and Au-5 nm/TA, respectively (Fig. 5a–c). In comparison with the normal control- and DSS control-treated animals, the Au-5 nm/TA-treated mice displayed a reduction of Roseburia, Ruminococcaceae, and Odoribacter, which are short chain fatty acid (SCFA)-producing bacteria present at decreased abundance in IBD patients [27]. This again suggests a potential unfavorable effect resulting from the oral administration of AuNPs on the gut bacterial populations.

Compared to normal control animals, those taxa altered by the DSS treatment were limited to the levels of genus and species, and were not closely related in the cladogram (Additional file 1: Figure S4). As shown in Fig. 5d, e, the taxonomic branch of *Lactobacillus gasseri*-*Lactobacillus*-Lactobacillaceae-Lactobacillales-Bacilli was underrepresented in the AuNP-treated mice compared with both normal control and DSS control-treated animals, indicating an adverse effect of oral administration of AuNPs against the beneficial genus *Lactobacillus*. Within the phylum of Bacteroidetes, the taxonomic branches of Bacteroidales-Bacteroidia-Bacteroidetes, and *Bacteroides acidifaciens*-*Bacteroidaceae*-Bacteroides, were overrepresented in the Au-5 nm/TA group compared with the DSS control group and the normal control group, respectively (Fig. 5d, e).

Since the oral administration of Au-5 nm/TA seemed to unfavorably alter mouse gut microbiota by decreasing the biodiversity and F/B ratio, as well as certain SCFA-producing bacteria and *Lactobacillus*, we hypothesize that AuNPs exerted anti-colitis activities through their anti-inflammatory rather than gut microbiota-modulating properties. There remains a lack of comprehensive understanding of the anti-inflammatory mechanism of AuNPs. To gain a better understanding of the mechanisms, the RAW264.7 cell line was utilized to depict typical interaction scenarios between AuNPs and inflammatory responses.

### Anti-inflammatory effects of AuNPs in lipopolysaccharides (LPS)-activated RAW264.7 cells

**Fig. 6:**
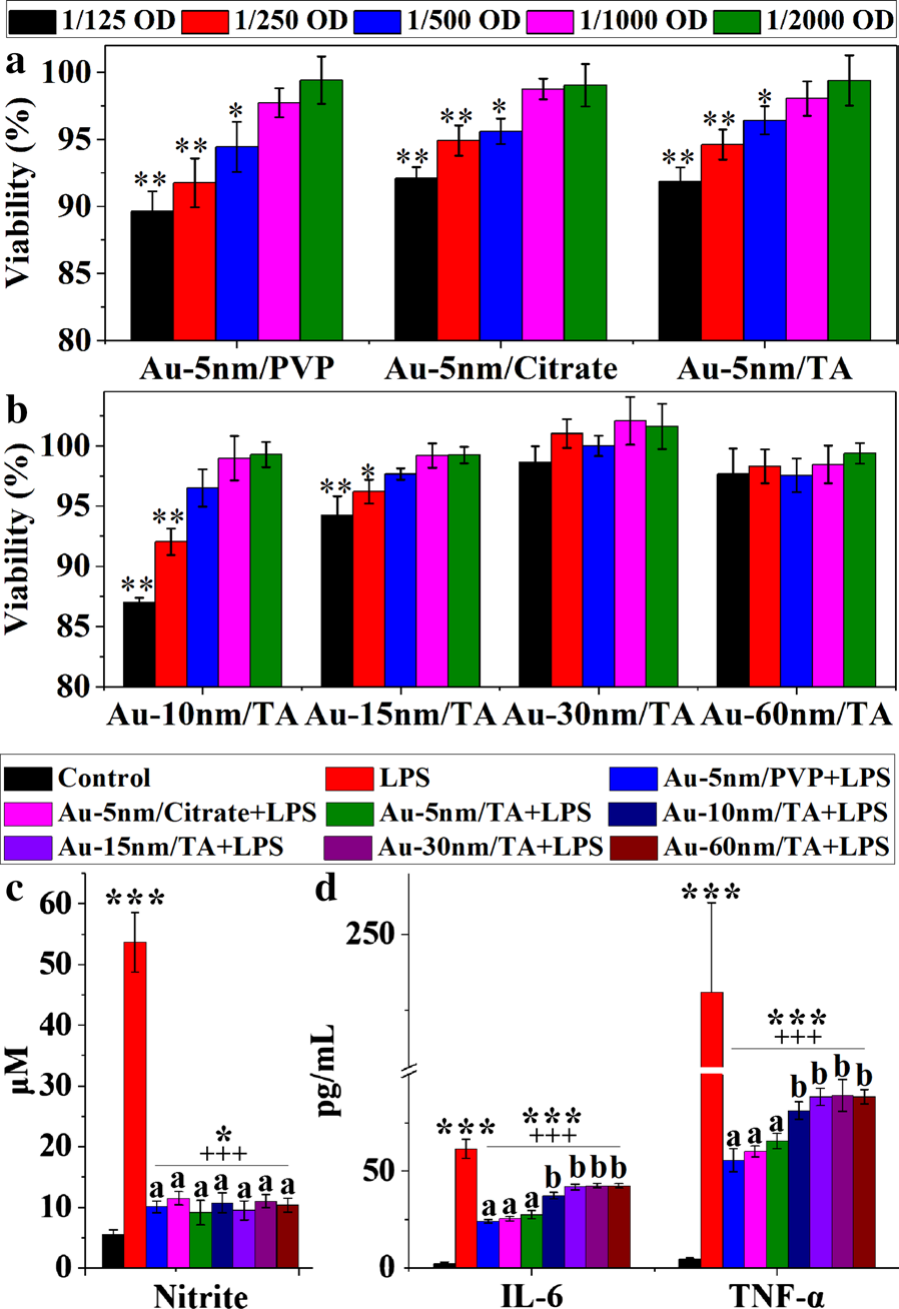
The biological effects of AuNPs on RAW264.7 cells: **a**, **b** cell viability measured by MTT assay (n = 6), with significance tested by one-way ANOVA with Dunnett’s post hoc test (*P < 0.05 and **P < 0.01, versus the control); **c**, **d** production of NO (n = 6), IL-6 (n = 3) and TNF-α (n = 3) following LPS activation, with asterisks and plus signs representing significant differences (***P < 0.001, *P < 0.05, ^+++^*P* < 0.001, Dunnett’s post hoc test following ANOVA) versus the normal control and the LPS control, respectively, and different lower-case letters marking significant differences (*P* < 0.05, ANOVA followed by LSD test) between AuNP-treated groups

To determine the highest non-cytotoxic concentrations of AuNPs, the methylthiazolyldiphenyl-tetrazolium bromide (MTT) test was performed 24 h after the treatment of RAW264.7 cells with various concentrations of AuNPs. At concentrations of 1/1000 OD or lower, AuNPs caused no statistically significant changes in macrophage viability (Fig. 6a, b). Au-5 nm/TA, Au-5 nm/PVP and Au-5 nm/Citrate displayed significant cytotoxicity at the concentration of 1/500 OD (Dunnett’s, *P* < 0.05), while TA-stabilized 10-nm AuNPs (Au-10 nm/TA) and Au-15 nm/TA showed cytotoxicity at concentrations above 1/250 OD (Dunnett’s, *P* < 0.05). TA-stabilized 30-nm and 60-nm AuNPs (Au-30 nm/TA and Au-60 nm/TA) induced no cytotoxicity at a concentration as high as 1/125 OD. These results suggest a size dependence in cellular toxicity of these AuNPs. As previously reported [28–30], AuNPs with sizes < 10 nm are generally more cytotoxic than those larger ones possibly due to their abilities to enter the cell nucleus.

Inductively coupled plasma mass spectrometry (ICP-MS) analysis of cells treated with 1/1000 OD of AuNPs revealed an increased absorption of 5-nm AuNPs coated with PVP compared to those coated with citrate and TA (LSD, *P* < 0.05) (Table 3). A size dependence in cellular uptake of TA-coated AuNPs was observed with Au-15 nm/TA and Au-30 nm/TA showing greater proportions of absorption (LSD, *P* < 0.05) (Table 3).

**Table 3:** The absorption of AuNPs by RAW264.7 cells

AuNPs	Dose (ng/10^7^ cells)	Absorptivity^a^	Au contents (ng per mg protein)^1^
Au-5 nm/PVP	231.3	14.6 ± 3.2%^b^	11.7 ± 1.7^ab^
Au-5 nm/Citrate	231.3	11.6 ± 2.6%^c^	9.3 ± 2.1^bc^
Au-5 nm/TA	231.3	9.5 ± 2.5%^c^	7.6 ± 1.8^c^
Au-10 nm/TA	202.3	12.9 ± 3.6%^bc^	9.1 ± 2.3^bc^
Au-15 nm/TA	187.0	20.1 ± 3.2%^a^	13.3 ± 2.6^a^
Au-30 nm/TA	163.7	19.8 ± 4.3%^a^	11.4 ± 2.3^ab^
Au-60 nm/TA	143.5	17.8 ± 3.6%^ab^	9.0 ± 1.5^bc^

^1^Values are means ± standard deviations. Means in a column without a common superscript letter (a, b and c) are statistically different (ANOVA followed by LSD test, *P* < 0.05)

LPS is a primary bacterial toxin that is used to initiate the inflammatory cascade leading to intestinal injury. To evaluate the anti-inflammatory activities of AuNPs, RAW264.7 cells were pretreated with 1/1000 OD of the particles for 5 h before stimulation with LPS and incubation for another 20 h. LPS significantly increased the production of nitric oxide (NO), IL-6 and TNF-α by the macrophages (Dunnett’s, *P* < 0.001), and these effects were markedly attenuated by pretreatments with AuNPs (Dunnett’s, *P* < 0.001) (Fig. 6c, d). All AuNPs had similar activities in attenuating the LPS-stimulated production of NO; however, the 5-nm AuNPs with the three types of coatings displayed uniformly superior anti-inflammatory activities in suppressing the production of the proinflammatory cytokines of IL-6 and TNF-α, when compared to the larger-sized TA-coated AuNPs (LSD, *P* < 0.05).

### AuNPs attenuated Toll-like receptor 4 (TLR4)- and reactive oxygen/nitrogen species (ROS/RNS)-mediated inflammatory signaling

**Fig. 7:**
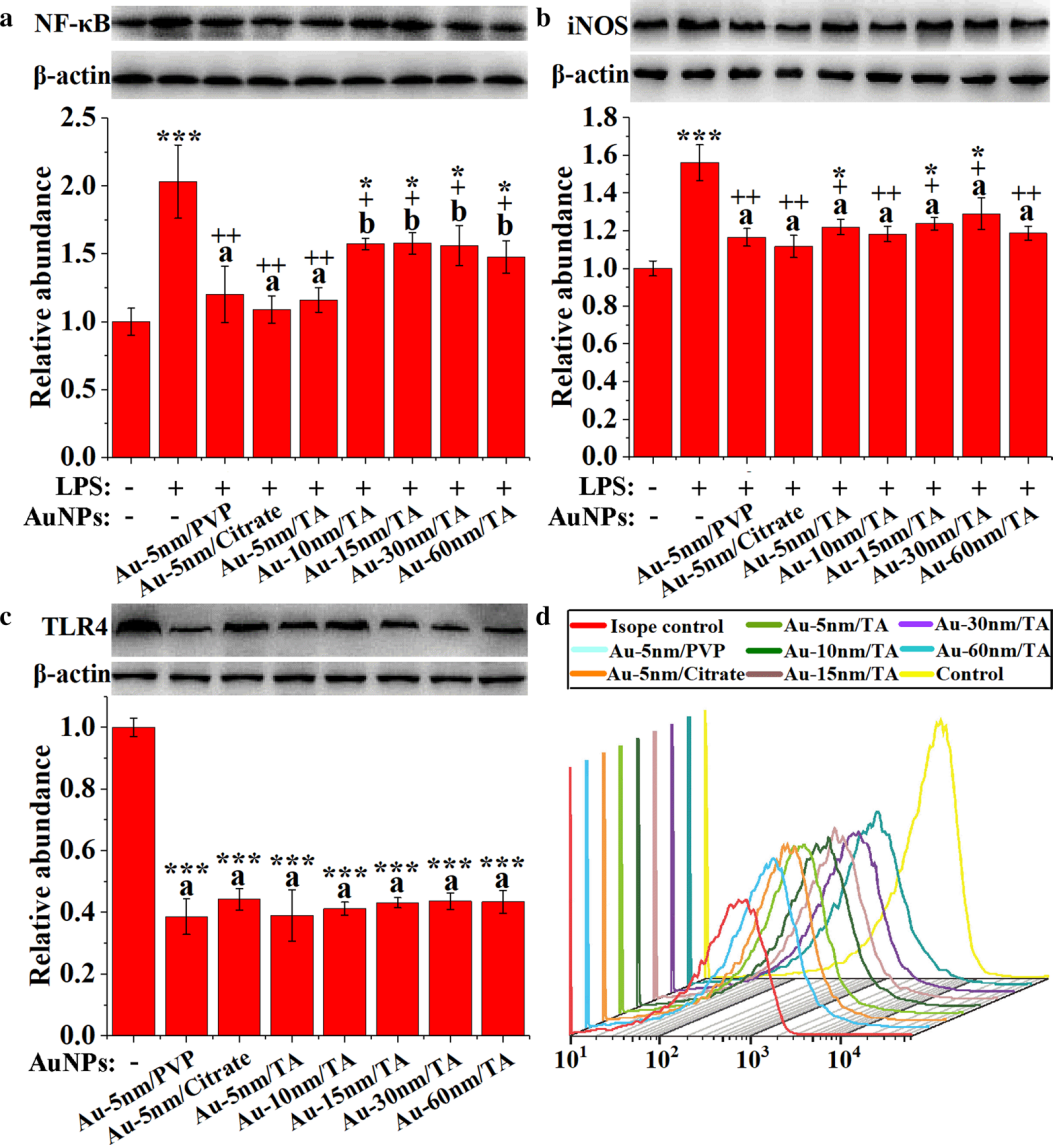
Western blotting analysis of the expression of **a** NF-κB p65 (n = 3), **b** iNOS (n = 3), and **c** TLR-4 (n = 3), with asterisks and plus signs representing significant differences (***P < 0.001, *P < 0.05, ^+++^*P* < 0.001, ^++^*P* < 0.01, ^+^*P* < 0.05, Dunnett’s post hoc test following ANOVA) versus the normal control and the LPS control, respectively, and different lower-case letters marking significant differences (*P* < 0.05, ANOVA followed by LSD test) between AuNP-treated groups; **d** flow cytometric analysis of cell surface TLR4

NF-κB is a key transcription factor dictating the production of proinflammatory cytokines, and its activation by LPS stimulation for 1 h was examined using Western blot analysis (Fig. 7a). Pretreatment of RAW264.7 cells with AuNPs for 5 h, resulted in significant inhibition of the LPS-stimulated increase of NF-κB p65 protein (Dunnett’s, *P* < 0.05). The 5-nm AuNPs with three different coatings uniformly exhibited higher activities than the larger TA-coated AuNPs (LSD, *P* < 0.05), which aligned well with the results of IL-6 and TNF-α in Fig. 6d. These results suggest that AuNPs inhibited the expression of proinflammatory cytokines through blocking NF-κB activation in RAW264.7 cells.

The results of Western blot analysis (Fig. 7b) showed that the expression of inducible NO synthase (iNOS) was increased by 24-h LPS stimulation (Dunnett’s, *P* < 0.001), and this was significantly reduced by the 5-h pretreatments with AuNPs (Dunnett’s, *P* < 0.05). Similar with the results of NO production (Fig. 6c), AuNPs with different coatings and sizes showed equivalent activities to block the LPS-induced expression of iNOS. These results suggest that AuNPs attenuated NO production by down-regulating the expression of iNOS in RAW264.7 cells.

LPS is recognized by macrophages via the TLR4/myeloid differentiation factor 2 (MD-2) receptor complex on the cell surface. Cell surface membrane and cell lysate levels of TLR4 protein were determined using flow cytometry and Western blot analysis, respectively. As shown in Fig. 7c, d, the treatment of RAW264.7 cells with AuNPs for 5 h greatly decreased the levels of TLR4 protein in cell lysates and membranes surfaces (Dunnett’s, *P* < 0.001), suggesting that AuNPs desensitized RAW264.7 cells to LPS. There were no differences in the reduction of TLR4 protein between AuNPs of different coatings or different sizes, which was similar to the findings for iNOS expression and NO production. These results suggest that AuNPs exerted their anti-inflammatory effects at least in part via modulation of the LPS/TLR4 signaling pathway.

**Fig. 8:**
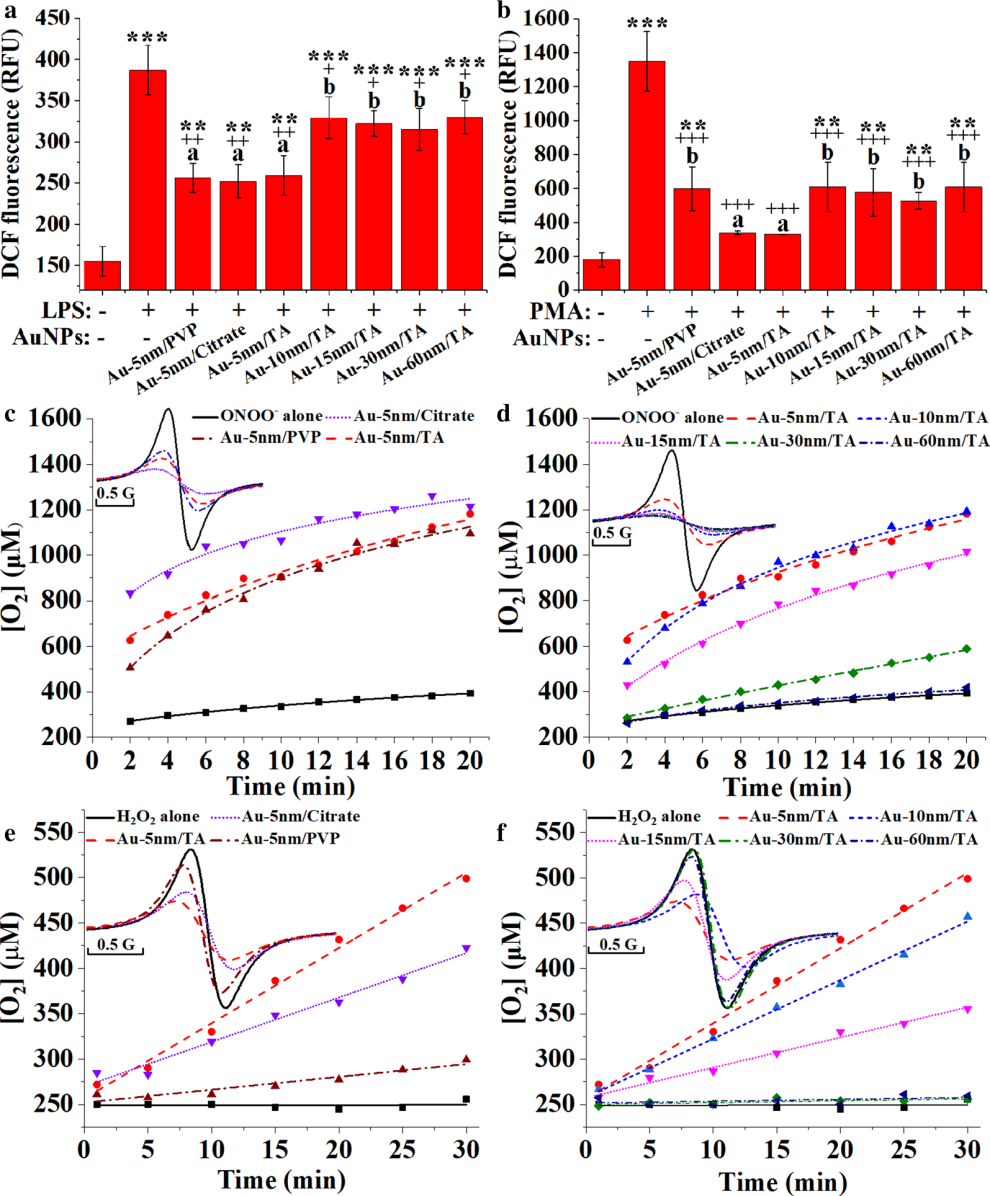
Effects of AuNPs on oxidant production in RAW264.7 cells stimulated by **a** LPS and **b** PMA, with asterisks and plus signs representing significant differences (***P < 0.001, **P < 0.01, ^+++^*P* < 0.001, ^++^*P* < 0.01, ^+^*P* < 0.05, Dunnett’s post hoc test following ANOVA) versus the normal control and the LPS/PMA control, respectively, and different lower-case letters marking significant differences (*P* < 0.05, ANOVA followed by LSD test) between AuNP-treated groups; the disproportionation of **c**, **d** peroxynitrite and **e**, **f** hydrogen peroxide with or without the presence of AuNPs, with the ESR spectra of ^15^N-PDT shown in the insets

NO and superoxide are simultaneously produced in LPS-activated macrophages, and they readily react to form a strong oxidant, peroxynitrite [31]. As shown in Fig. 8a, the 5-h pretreatment of RAW264.7 cells with the AuNPs significantly attenuated the arise in dichlorodihydrofluorescein (DCF) fluorescence in response to 1-h LPS stimulation (Dunnett’s, *P* < 0.05) and suggested that AuNPs prevented the LPS-induced burst of intracellular peroxynitrite. The three different coated 5-nm AuNPs were uniformly superior in suppressing the formation of peroxynitrite, when compared to the larger TA-coated AuNPs (LSD, *P* < 0.05). These results contrasted with the results for TLR4 protein reduction (Fig. 7c, d); thus, the attenuation of the LPS/TLR4 signaling cannot completely explain the prevention of LPS-mediated oxidative burst by AuNPs.

The disproportionation of peroxynitrite into the weak oxidants, O_2_ and nitrite, occurs under physiological conditions as a detoxification mechanism [32]. Peroxynitrite has a half-life over 1 s under physiological conditions [33], so it is stable enough to diffuse into endosomes for microbial killing, as well as interacting with the endocytosed AuNPs. The results of electron spin resonance (ESR) oximetry in vitro (Fig. 8c, d) showed that AuNPs catalyzed peroxynitrite disproportionation in a size- and coating-dependent manner. The catalytic activities of 5-nm AuNPs with three coatings were uniformly higher than those of Au-15 nm/TA, Au-30 nm/TA and Au-60 nm/TA, which was in accordance with the prevention of LPS-induced oxidative burst (Fig. 8a), NF-κB activation (Fig. 7a), and cytokine production (Fig. 6d) by AuNPs. These results suggest that the catalytic detoxification of peroxynitrite may account for the size-dependency of anti-inflammatory activities of AuNPs in LPS-stimulated macrophages.

When superoxide is generated without the simultaneous production of NO during macrophage respiratory burst that is elicited by certain non-LPS stimuli, such as phorbol 12-myristate 13-acetate (PMA), the short-lived radical readily transforms into hydrogen peroxide, which is a relatively stable and highly diffusible oxidant regulating inflammatory activation [34]. AuNPs can catalyze hydrogen peroxide disproportionation under physiological conditions [35], which confers their antioxidant properties. As shown in Fig. 8b, the 5-h pretreatment of macrophages with AuNPs effectively prevented the arise in DCF fluorescence in response to PMA stimulation for 1 h (Dunnett’s, *P* < 0.001), suggesting that AuNPs could suppress the PMA-induced oxidative burst. In addition, Au-5 nm/TA and Au-5 nm/Citrate were shown to display significantly greater antioxidative activities than the other AuNPs (LSD, *P* < 0.05). The results of ESR oximetry showed that Au-5 nm/TA and Au-5 nm/Citrate exhibited better catalase-mimetic activities than Au-5 nm/PVP, Au-15 nm/TA, Au-30 nm/TA and Au-60 nm/TA in vitro (Fig. 8e, f). Thus, it appears that AuNPs may play an anti-inflammatory role in non-LPS-activated macrophages by catalyzing the detoxification of hydrogen peroxide.

From the above results one can infer that AuNPs attenuate the inflammatory responses via reduction of the LPS receptor and catalytic detoxification of peroxynitrite and hydrogen peroxide. The inhibition of LPS-induced NO production and iNOS expression by AuNPs may depend on TLR4 protein reduction alone, while the attenuation of proinflammatory cytokine production and NF-κB activation by AuNPs appear to associate with both TLR4 protein reduction and ROS/RNS detoxification.

### Summary

The 5-nm AuNPs coated with PVP and citrate were more efficient than the TA-coated AuNP as anti-colitis and anti-inflammatory agents in a mouse model of ulcerative colitis in this study, which can be explained by the differences in their bioavailabilities and peroxynitrite-detoxifying activities. Au-5 nm/PVP had a higher bioavailability than Au-5 nm/TA and Au-5 nm/Citrate in both mice and macrophages, while Au-5 nm/Citrate had a greater peroxynitrite-detoxifying activity than Au-5 nm/TA and Au-5 nm/PVP in vitro.

PVP is a kind of amphiphilic biopolymer, while TA and citrate are hydrophilic organics; therefore, Au-5 nm/PVP should possess higher surface hydrophobicity than Au-5 nm/TA and Au-5 nm/Citrate. Surface hydrophobicity is a critical factor governing cell-nanoparticle interactions, and its effect is even stronger than that of surface charge [36]. Nanoparticles with more hydrophobicity are generally internalized better by mammalian cells due to their higher affinity for the cell membrane [37]. Therefore, the higher surface hydrophobicity of Au-5 nm/PVP particles may explain their enhanced absorption in mice and macrophages compared to Au-5 nm/TA and Au-5 nm/Citrate.

Among the differently sized TA-coated AuNPs, Au-15 nm/TA had the highest bioavailability in mice and macrophages. Schleh et al. reported similar results that oral administration of AuNPs in the size range of 5–200 nm were most bioavailable at the size of 18 nm in rats [17]. AuNPs enter cells primarily via receptor-mediated endocytosis (RME) pathways [28]. The optimal particle size for RME is theoretically at 54–60 nm in diameter for spherical particles [38]. Au-15 nm/TA gave the smallest hydrodynamic size of 58.8 nm in 10% FBS-supplemented DMEM, and thus seem to have an aggregate size most suitable for RME in biological fluids. This might explain the reason for the highest bioavailability of Au-15 nm/TA among TA-coated AuNPs. However, we observed no size effect in the anti-colitis and anti-inflammatory activities of TA-coated AuNPs.

A reduction of cell surface TLR4 protein by AuNPs in macrophages has never been shown before, and this might be the result of co-endocytosis of AuNPs with membrane TLR4. Husebye et al. found that upon binding to LPS, the TLR4/MD-2 complex on the cell membrane of macrophages would be endocytosed via RME and subsequently degraded in late endosomes/lysosomes as the rapid mechanism of LPS desensitization [39]. Liu et al. reported that LPS stimulation promptly accelerated the endocytic uptake of AuNPs by RAW264.7 cells [40]. Tsai et al. discovered that the AuNP-treatment induced a rapid translocation of membrane TLR9 into the phagosomes in Raw264.7 cells [13]. We thus hypothesize that AuNPs were internalized by macrophages and this was accompanied by an endocytic translocation of membrane TLR4 leading to the rapid LPS desensitization.

AuNPs were found as a novel peroxynitrite decomposition catalyst in this study. Peroxynitrite is a relatively stable, diffusible, and highly reactive oxidant contributing to a host of pathologies, so peroxynitrite decomposition catalysts, which mainly fall into three categories including iron/manganese metalloporphyrin, bis(hydroxyphenyl)dipyrromethenes and metallocorroles, have attracted growing interest in the treatment of disease [41]. To the best of our knowledge, metallic nanoparticles have not been shown to have a peroxynitrite decomposing activity. Our results might inspire potential clinical applications of noble metal nanoparticles in peroxynitrite-associated pathologies such as inflammatory and neurodegenerative diseases, type I diabetes, and myocardial infarction.

After ingestion, nanoparticles reach the intestine and encounter the gut microbiome. The interactions of nanoparticles with the gut microbiome may induce a number of effects that have consequences on human health. Recently, the interactions of silver, titanium dioxide, silica dioxide, zinc oxide, and carbon nanoparticles with gut microbiota in vivo have been reported, however, few studies have focused on the effects of AuNPs on gut microbial profiles [42, 43]. This study demonstrated that AuNPs reduced the F/B ratio and probiotic *Lactobacillus* abundances, which is in line with the results of silver nanoparticles as reported by Chen et al. [43]. Hydrogen peroxide production by probiotic bacteria has been supposed to contribute to the maintenance of a normal and homeostatic microbiota [44]. Considering their catalase-mimetic activities, AuNPs might induce gut dysbiosis by catalyzing intraluminal hydrogen peroxide decomposition. Additional studies are needed to further evaluate the mechanism for AuNPs to decrease *Lactobacillus* in the gut microbiome.

## Conclusions

In conclusion, the attenuation of colonic and systemic inflammation should be the key underlying mechanism for the anti-colitis effect of oral administration of AuNPs in mice. Specifically, oral administration of AuNPs unfavorably altered mouse gut microbiota. AuNPs could exert anti-inflammatory effect in macrophages by reduction of the cell surface LPS receptor and catalytic detoxification of peroxynitrite and hydrogen peroxide. AuNPs can serve as a new paradigm for treating IBD and other ROS/RNS-associated diseases, but precautions are needed to overcome their potentially negative impact on gut microbiota.

## Methods

### Source and characterization of AuNPs

Au-5 nm/TA, Au-10 nm/TA, Au-15 nm/TA, Au-30 nm/TA and Au-60 nm/TA were provided in 0.1 mM phosphate buffered saline by CytoDiagnostics (Burlington, Canada), and were quantified by using the OD at their SPR wavelengths. As per manufacturer, the stock solutions (OD = 1) of Au-5 nm/TA, Au-10 nm/TA, Au-15 nm/TA, Au-30 nm/TA and Au-60 nm/TA contained 69.4, 60.7, 56.1, 49.1, and 43.0 μg gold/mL, respectively. Au-5 nm/Citrate and Au-5 nm/PVP were supplied in water at the concentration of 1 mg/mL by nanoComposix, Inc. (CA, USA). The endotoxin levels in these commercial preparations were determined using the Pierce LAL Chromogenic Endotoxin Quantitation Kit (Thermo Scientific, CA, USA) as per manufacturer’s recommendations. Particle size was characterized by DLS, using a Malvern Zetasizer Nano ZS instrument (Herrenberg, Germany) equipped with a 633 nm He–Ne laser at 25 °C, and by TEM, using a JEOL JEM 2100 FEG instrument (Tokyo, Japan) at an accelerating voltage of 200 kV. Zeta potential was measured in pre-rinsed folded capillary cells on the Malvern Zetasizer Nano ZS instrument at 25 °C.

### Reagents

MTT, hydrogen peroxide, peroxynitrite, phenylmethanesulfonyl fluoride (PMSF), o-dianisidine dihydrochloride, LPS from *Escherichia coli* O55:B5, tGSH assay kit, and protease inhibitor cocktail and radioimmunoprecipitation assay (RIPA) lysis buffer were purchased from Sigma-Aldrich Co. (Shanghai, China). DSS, molecular weight 36–50 kDa, was provided by Yisheng Biotechnology Co., Ltd. (Shanghai, China). The Pierce BCA protein assay kit, 2′,7′-DCF diacetate, the enhanced chemiluminescence (ECL) detection kit, and sandwich enzyme-linked immunosorbent assay (ELISA) kits for mouse IL-6 and TNF-α, DMEM, Dulbecco’s phosphate-buffered saline (DPBS) and Hank’s balanced salt solution (HBSS) were obtained from ThermoFisher Scientific (CA, USA). PMA, rabbit anti-mouse NF-kB p65 polyclonal antibody (ab32536), horseradish peroxidase (HRP)-conjugated goat anti-mouse IgG secondary antibody (ab6789), HRP-conjugated goat anti-rabbit IgG secondary antibody (ab205718), and mouse monoclonal anti-β-actin antibody (ab6276) were purchased from Abcam (Shanghai, China). 4-Hydroxy-2,2,6,6-tetramethylpiperidine-1-15*N*-oxyl (^15^N-PDT) was supplied by Cambridge Isotope Laboratories, Inc. (Andover, MA). Mouse monoclonal anti-TLR4 antibody (sc-293072), Alexa Fluor^®^ 647-conjugated anti-TLR4 antibody (sc-293072 AF647), and Alexa Fluor^®^ 647-conjugated mouse isotype control IgG_1_ (sc-24636) were purchased from Santa Cruz Biotechnology (Shanghai, China). Rabbit monoclonal anti-iNOS antibody (13120S) were provided by Cell Signaling Technology (Shanghai, China). FBS was purchased from ExCell Bio (Shanghai, China). Other reagents used were of analytical grade and commercially available.

### Animals

Male C57BL/6 mice (7–8 weeks old, weighing 21.5 ± 1.80 g) were purchased from Lukang Pharmaceutical Co. (Shandong, China). Animals were maintained in individual cages in an air-conditioned room (20–24 °C, 55–65% humidity) under a lighting regime of 12 h light:12 h dark. The mice had ad libitum access to a pelletized rodent chow (TROPHIC Animal Feed High-Tech Co., Ltd., Nantong, China) throughout the course of the experiments. Deionized water was used as drinking water. All experiments were performed ethically in accordance with the principles of the National Institutes of Health *Guide for the Care and Use of Laboratory Animals* and were approved by the Committee on the Ethics of Animal Experiments of Ocean University of China.

### Induction of colitis and treatment protocol

The DSS model is a well-established model for the induction of colitis and has been used as a screening tool for new drugs and to investigate their mechanism of action [45]. Following a 3-day acclimation period, mice were randomly divided into nine treatment groups (8 mice/group), comprising normal control, DSS control, DSS + Au-5 nm/Citrate, DSS + Au-5 nm/PVP, DSS + Au-5 nm/TA, DSS + Au-10 nm/TA, DSS + Au-15 nm/TA, DSS + Au-30 nm/TA, and DSS + Au-60 nm/TA groups. The normal control group had ad libitum *access to* drinking water for 8 days (Fig. 1a). Experimental colitis was induced by the administration of 3% (w/v) DSS in the drinking water of mice with ad libitum access for 5 days, which was then followed by ad libitum access to regular drinking water for the remaining 3 days. All groups of mice received daily intragastric intubations for 8 days, consisting of 200 μL drinking water for the normal and DSS control groups and 200 μL of 1/25 OD of the specified AuNPs for the gold-treated groups. The oral gavage dosages of AuNPs corresponded to 0.55, 0.55, 0.55, 0.49, 0.45, 0.39, and 0.34 μg gold/animal and were approximately equivalent to 25, 25, 25, 22, 20, 18, and 15 μg gold/kg bodyweight, for the Au-5 nm/Citrate, Au-5 nm/PVP, Au-5 nm/TA, Au-10 nm/TA, Au-15 nm/TA, Au-30 nm/TA or Au-60 nm/TA groups of mice, respectively.

### Evaluation of colitis

Food intake and individual animal body weights were recorded daily, and all mice were observed daily for clinical symptoms of colitis, including stool consistency and fecal blood [46]. Stool consistency was scored as follows: 0 = normal; 1 = moist/sticky stool; 2 = soft stool; 3 = diarrhea. Fecal blood was scored as follows: 0 = no blood; 1 = evidence of blood in stool or around anus; 2 = severe bleeding. Weight loss was scored as follows: 0 = no loss; 1 = 1–5%; 2 = 5–10%. To evaluate the severity of colitis, DAI was calculated as the sum of the scores of stool consistency, fecal blood, and body weight loss.

### Animal sacrifice and necropsy

At the end of the study, fecal samples were collected and stored at − 80 °C until gut microbiota compositional analysis. Blood was collected by eyeball enucleation for hematology and for serum cytokine level evaluations. Serum samples were prepared for cytokine production by allowing the whole blood to clot for about 30 min at room temperature followed by centrifugation of the clotted blood at 3000 rpm for 15 min at 4 °C. The blood samples for hematological analysis were immediately collected into tubes containing ethylenediamine tetra-acetic acid (EDTA), and white blood cells and lymphocytes in the samples were analyzed on a XN-9000 hematology analyzer (Sysmex, Kobe, Japan), with all hemolyzed or clotted samples discarded before analysis.

Animals were sacrificed after exsanguination. The colon was removed intact from the abdominal cavity, and the length of each colon from the ileo-cecal junction to the anus were measured at rest, without stretching. The proximal colon was then cut into small pieces (~ 1 cm long) for histological and biochemical analysis. Both kidneys were harvested and heated to dryness before being refluxed at 90 °C in aqua regia for 24 h. The gold concentrations in the digested kidney samples were measured by ICP-MS (Agilent Technologies, Beijing, China).

### Histopathology

For H&E staining, the proximal colonic tissues were fixed in 10% neutral buffered formaldehyde, desiccated, and embedded in paraffin. The paraffin embedded tissues were cut into thin sections (5 μm) and stained with H&E for histologic evaluations, using an OLYMPUS BX41 light microscope (Japan). Six randomly selected fields for each sample were imaged, and the histologic lesions in each field were scored, as described by Laroui et al., by an experienced pathologist from the Department of Clinical Laboratory at the Affiliated Hospital of Qingdao University [46]. A mean score of 6 fields was used as the histologic score for each mouse.

### MPO assay

The colonic MPO activity was measured according to the procedures described by Rodriguez-Palacios et al. [47]. Pre-weighed proximal colonic samples (stored for < 48 h at − 80 °C) were placed in a microfuge tube that contained 20 volumes of ice-cold potassium phosphate buffer (50 mM, pH 6.0) with 0.5% cetyltrimethylammonium bromide. The tissue was homogenized with ceramic beads, using a Bioprep-6 Homogenizer (Allsheng Instruments CO., Ltd, Hangzhou, China), and the homogenate was centrifuged at 4 °C for 5 min at 12,500 rpm. Ten microliter of the supernatant were loaded in each well of a pre-chilled ELISA plate, and 200 μL of the active substrate solution containing 0.2 mg/mL o-dianisidine hydrochloride and 0.001% hydrogen peroxide were then added to each well. The absorbance at 450 nm was read immediately and every 30 s afterwards for 5 min with a Synergy H4 hybrid microplate reader (Bio-Tek, Winooski, VT, USA) set to 28 °C. One unit of MPO activity was calculated as: MPO activity = ΔA450 ÷ 0.5 ÷ 0.0113 ÷ 0.05; where ΔA450 was the average of ΔA450_(t30–t0)_ (the absorbance difference from time 0 to 30 s) and ΔA450_(t60–t30)_ (the absorbance difference from time 30 to 60 s); 0.0113 is the MPO constant; 0.5 is for the time interval (i.e. 0.5 min), and 0.05 is the dilution factor of sample: CTAB lysis buffer (i.e. 50 mg:1 mL).

### Quantification of tGSH in colon tissues

Pre-weighed proximal colonic samples were added to 10 volumes of the 5% 5-sulfosalicylic acid solution, and then the homogenization was carried out in a homogenizer tube containing ceramic beads using a Bioprep-6 Homogenizer. The homogenate was centrifuged at 10,000 rpm for 5 min at 4 °C, and the supernatant was used as the tissue extract. The level of tGSH in the tissue extract was determined using the tGSH assay kit as per the manufacturer’s instructions.

### Cytokine assay in serum and colon tissue

Pre-weighed proximal colonic samples were added to 25 volumes of RIPA lysis buffer containing PMSF (1 mM) and protease and phosphatase inhibitor cocktails, and then the homogenization was carried out in a homogenizer tube containing ceramic beads using a Bioprep-6 Homogenizer. The homogenate was centrifuged (10,000 rpm, 5 min, 4 °C), and the supernatant was used as the tissue extract. The concentrations of IL-6 and TNF-α in tissue extracts and serum samples were determined using ELISA kits as per the manufacturer’s instructions.

### Gut microbiota analysis

Considering the cost, a total of 15 fecal samples were randomly chosen from the normal control group (n = 5), the DSS control group (n = 5), and the Au-5 nm/TA group (n = 5) for gut microbiota analysis. Total bacterial DNA was extracted using the QIAamp DNA Stool Mini Kit (Qiagen, Hilden, Germany) as per the manufacturer’s instructions. The integrity, purity, fragment size and concentration of the extracted DNA were determined by 1% agarose gel electrophoresis. The primers 515F (5′-GTGYCAGCMGCCGCGGTAA-3′) and 907R (5′-CCYCAATTCMTTTRAGTTT-3′) were used to amplify the V4–V5 regions of 16S rRNA gene. The 16S rRNA tag-encoded high-throughput sequencing was performed on an Illumina HiSeq 2500 high-throughput sequencer (Illumina, San Diego, USA) at Novogene Bioinformatics Technology Co., Ltd. (Beijing, China). Paired-end reads were assigned to samples based on their unique barcode, and were truncated by eliminating the barcode and primer sequence. The paired-end reads were merged using FLASH. Quality filtering of the raw sequencing data was carried out to get high-quality clean tags, which were then analyzed with the QIIME (Quantitative Insights into Microbial Ecology) software package using default settings [48]. Sets of sequences with 97% similarity were assigned as an OTU using UPARSE pipeline, and a representative sequence for each OUT was employed to annotate the taxonomic information in Mothur with the SILVA SSU Ref database [49, 50]. The α-diversity metric ACE, as well as PCA of β-diversity utilizing the unweighted UniFrac, was calculated by QIIME software and displayed using R software. The LEfSe method was employed to compare taxa at species or higher levels among groups [51].

### Cellular experiments

RAW264.7 cells were originally obtained from the Cell Bank of the Chinese Academy of Sciences (Shanghai, China) and were maintained routinely in DMEM medium supplemented with 10% FBS at 37 °C in a 5% CO_2_ atmosphere. To evaluate cellular toxicity, cells were seeded at a density of 1 × 10^5^ cells/well in 96-well plates and cultured for 24 h in complete media. The cells were rinsed three times with DPBS and incubated in complete media with and without AuNPs for another 24 h. Cell viability was measured by the MTT assay. Briefly, the cells were incubated with 0.5 mg/mL MTT in fresh medium for 4 h, and the resulted formazan crystals were dissolved with DMSO, followed by measuring the absorbance at 570 nm on a Synergy H4 hybrid microplate reader.

To assay the production of cytokines and NO, RAW264.7 cells seeded into a 48-well plate (5.0 × 10^5^ per well) were cultured for 24 h in complete media. Cells were incubated in complete media with and without AuNPs (OD = 0.001) for 5 h. Cells were rinsed with DPBS before the cells were incubated for 20 h in media supplemented with or without LPS (1 μg/mL). The cell culture supernatant was then harvested and stored at − 20 °C before analysis.

To measure NO production, the nitrite levels in cell culture supernatants were colorimetrically determined using Griess reagent. Briefly, the culture supernatant (100 μL) was mixed with an equal volume of freshly prepared Griess reagent (1% sulphanilamide, 0.1% N-1-naphthyl ethylenediamine, and 5% phosphoric acid) for 10 min in a 96-well plate, and the absorbance at 540 nm was then measured on a plate reader (Bio-Tek, Winooski, VT, USA). Nitrite concentration was calculated from a sodium nitrite standard curve. The concentrations of IL-6 and TNF-α cell in culture supernatants were determined using ELISA kits as per the manufacturer’s instructions.

To determine the production of ROS/RNS, cells were seeded into 48-well plates (5.0 × 10^5^ per well) and cultured for 48 h. The cells were incubated with or without AuNPs (OD = 0.001) for 5 h, rinsed, and incubated for 1 h in media supplemented with or without LPS (1 μg/mL) or PMA (1.25 μg/mL). The cells were then exposed to 2′,7′-DCF diacetate (20 μM), for 30 min to allow the dye to diffuse into the cells. The excess dye was removed, and cells were washed twice with DPBS prior to measurement of absorbance with a Synergy H4 hybrid microplate reader set at 485-nm excitation and 535-nm emission.

Flow cytometry and Western blotting were performed to measure TLR4 protein levels. For these experiments, RAW264.7 cells were seeded into 6-well plates (1.0 × 10^6^ per well) and were cultured for 48 h in complete media. The cells were rinsed and incubated with or without AuNPs (OD = 0.001) for 5 h. For flow cytometry, cells were rinsed and detached with EDTA. The cells were then incubated with Alexa Fluor^®^ 647-conjugated anti-TLR4 or the matched isotype control IgG_1_ (1 μg/10^6^ cells) for 30 min on ice, washed and analyzed on a BD Accuri C6 flow cytometer (BD Biosciences, Beijing, China). For Western blotting, cells were rinsed and lysed in RIPA buffer containing PMSF (1 mM) and protease and phosphatase inhibitor cocktails at 4 °C for 30 min, and after centrifugation at 13,000 r/min for 15 min at 4 °C, the supernatant was used as whole cell lysate for further analysis.

For Western blotting analysis of NF-κB p65 and iNOS, RAW264.7 cells were rinsed and stimulated with or without LPS (1 μg/mL) for 1 h (NF-κB p65) or 24 h (iNOS). Cells were then lysed in RIPA buffer containing PMSF (1 mM) and protease and phosphatase inhibitor cocktails at 4 °C for 30 min, and after centrifugation at 13,000 r/min for 15 min at 4 °C, the supernatant was used as whole cell lysate for further analysis.

To measure the uptake of AuNPs, cells seeded into 6-well plates (1.0 × 10^6^ per well) were cultured for 24 h before incubation with or without AuNPs (OD = 0.001) for another 24 h. The cells were then washed three times and digested with aqua regia at room temperature for 6 h. The gold concentrations in the cellular digests were determine by ICP-MS.

### Western blotting

The protein concentration in whole cell lysate was measured using the Pierce BCA protein assay kit as per the manufacturer’s instructions. After cell lysate samples were denatured in SDS-loading buffer, they were separated by SDS–PAGE gel electrophoresis and blotted onto a PVDF membrane. After being blocked for 1 h at room temperature with TBST [10 mM tris (pH 7.5), 150 mM NaCl, and 0.05% Tween 20] containing 5% fat-free dried milk, the membrane was incubated with anti-TLR4 (1:200 dilution), anti-NF-κB p65 (1:8000 dilution), anti-β-actin (1:5000 dilution), or iNOS (1:200 dilution) overnight at 4 °C. Immune complexes were incubated for 1 h at room temperature with HRP-conjugated secondary antibodies (1:5000 dilution), and were then washed with TBST for three times before being visualized using an ECL detection kit on a Tanon-5200 Multi image analyzer (Tanon Science & Technology Co., Ltd., Shanghai, China).

### ESR oximetry

ESR oximetry with spin label ^15^N-PDT was used to determine the catalase and peroxynitrite decomposition activity of AuNPs. The reaction mixture contains 0.2 mM ^15^N-PDT, 5 mM hydrogen peroxide, and AuNPs (OD = 0.8) for catalase activity, and 0.2 mM ^15^N-PDT, 11 mM peroxynitrite, and AuNPs (OD = 0.1) for peroxynitrite decomposition. ESR spectra recording began 1 min after sample mixing on a Bruker EMX ESR spectrometer with the following settings: 1 mW microwave power, 0.05 G field modulation, and 2 G scan range. The concentration of O_2_ produced in the reaction was estimated using the peak to peak line width of the ^15^N-PDT lower field ESR line according to Liu et al. (Additional file 1: Figure S1) [52].

### Statistical analysis

Statistical analyses were performed with SPSS software version 19.0 (SPSS, Inc., Chicago, USA) and OriginPro 7.0 software (OriginLab Co., Northampton, USA). One-way analysis of variance (ANOVA) or Wilcoxon signed-rank test was used to compare the mean differences. Dunnett’s test (two-tailed) was used for post hoc pairwise comparisons to the normal control or the DSS control. LSD test was used for post hoc pairwise comparisons between DSS-treated groups. Significant differences were defined at probability values (*P*-values) of *P* < 0.05, *P* < 0.01 and *P* < 0.001.

## Additional file

**Additional file 1.** **Figure S1.** The linear relationship between line width of ESR spectra and O_2_ concentration in solutions. **Figure S2.** Transmission electron microscopy images of AuNPs. **Figure S3.** Rarefaction curves calculated for each sample. **Figure S4.** Taxonomic cladogram generated from LEfSe analysis showing significant difference in microbiota profile of the groups of normal control and DSS control.

## Abbreviations

ACE: abundance-based coverage estimator
DAI: Disease Activity Index
DCF: dichlorodihydrofluorescein
ESR: electron spin resonance
F/B: ratio of firmicutes to bacteroidetes
ICP-MS: inductively coupled plasma mass spectrometry
IL-6: interleukin-6
iNOS: inducible nitric oxide synthase
LEfSe: linear discriminant analysis effect size
LPS: lipopolysaccharides
LSD: least significant difference
MTT: methylthiazolyldiphenyl-tetrazolium bromide
MPO: myeloperoxidase
OD: optical density
NF-κB: nuclear factor kappa beta
NO: nitric oxide
OUT: Operational Taxonomic Units
PCA: principal component analysis
SCFA: short chain fatty acid
SPR: surface plasmon resonance
TA: tannic acid
TEM: transmission electron microscopy
tGSH: total glutathione
TLR4: Toll-like receptor 4
TNF-α: tumor necrosis factor-α
UniFrac: unique fraction
