# Supplementary material for: Orally administered gold nanoparticles protect against colitis by attenuating Toll-like receptor 4- and reactive oxygen/nitrogen species-mediated inflammatory responses but could induce gut dysbiosis in mice
Source: J Nanobiotechnology. 2018 Nov 1;16:86. doi: 10.1186/s12951-018-0415-5 (PMC6211593; doi:10.1186/s12951-018-0415-5)
Supplement: Supplementary file 1 — Additional file 1. Figure S1. The linear relationship between line width of ESR spectra and O2 concentration in solutions. Figure S2. Transmission electron microscopy images of AuNPs. Figure S3. Rarefaction curves calculated for each sample. Figure S4. Taxonomic cladogram generated from LEfSe analysis showing significant difference in microbiota profile of the groups of normal control and DSS control. [file 12951_2018_415_MOESM1_ESM.docx]

Additional Information

Orally administered gold nanoparticles protect against colitis in mice by attenuating Toll-like receptor 4- and reactive oxygen/nitrogen species-mediated inflammatory responses but could induce gut dysbiosis

Suqin Zhu ^1^, Xiumei Jiang ^2^, Mary D. Boudreau ^3^, Guangxin Feng ^1^, Yu Miao ^4^, Shiyuan Dong ^1^, Haohao Wu ^1^*, Mingyong Zeng ^1^*, Jun-Jie Yin ^2^

^1^ College of Food Science and Engineering, Ocean University of China, 5 Yushan Road, Qingdao, Shandong Province, 266003, China. Emails: [suqin.zhu.ouc@gmail.com](mailto:suqin.zhu.ouc@gmail.com) (Zhu, S.); [1604295925@qq.com](mailto:1604295925@qq.com) (Feng, G.); [dongshiyuan@ouc.edu.cn](mailto:dongshiyuan@ouc.edu.cn) (Dong, S.); [wuhaohao@ouc.edu.cn](mailto:wuhaohao@ouc.edu.cn) (Wu, H.); [mingyz@ouc.edu.cn](mailto:mingyz@ouc.edu.cn) (Zeng, M.)

^2^ Division of Analytical Chemistry, Office of Regulatory Science, Center for Food Safety and Applied Nutrition, U.S. Food and Drug Administration, College Park, MD 20740, USA. [Xiumei.Jiang@fda.hhs.gov](mailto:Xiumei.Jiang@fda.hhs.gov) (Jiang, X); [JunJie.Yin@fda.hhs.gov](mailto:JunJie.Yin@fda.hhs.gov) (Yin, JJ)

^3^ Division of Biochemical Toxicology, National Center for Toxicological Research, U.S. Food and Drug Administration, Jefferson 72079, AR, USA. [Mary.Boudreau@fda.hhs.gov](mailto:Mary.Boudreau@fda.hhs.gov) (Boudreau, M. D.)

^3^ Department of Clinical Laboratory, The Affiliated Hospital of Qingdao University, Qingdao, Shandong Province, 266003, China. [420724501@qq.com](mailto:420724501@qq.com) (Miao, Y.)

* Authors to whom correspondence should be addressed.

**
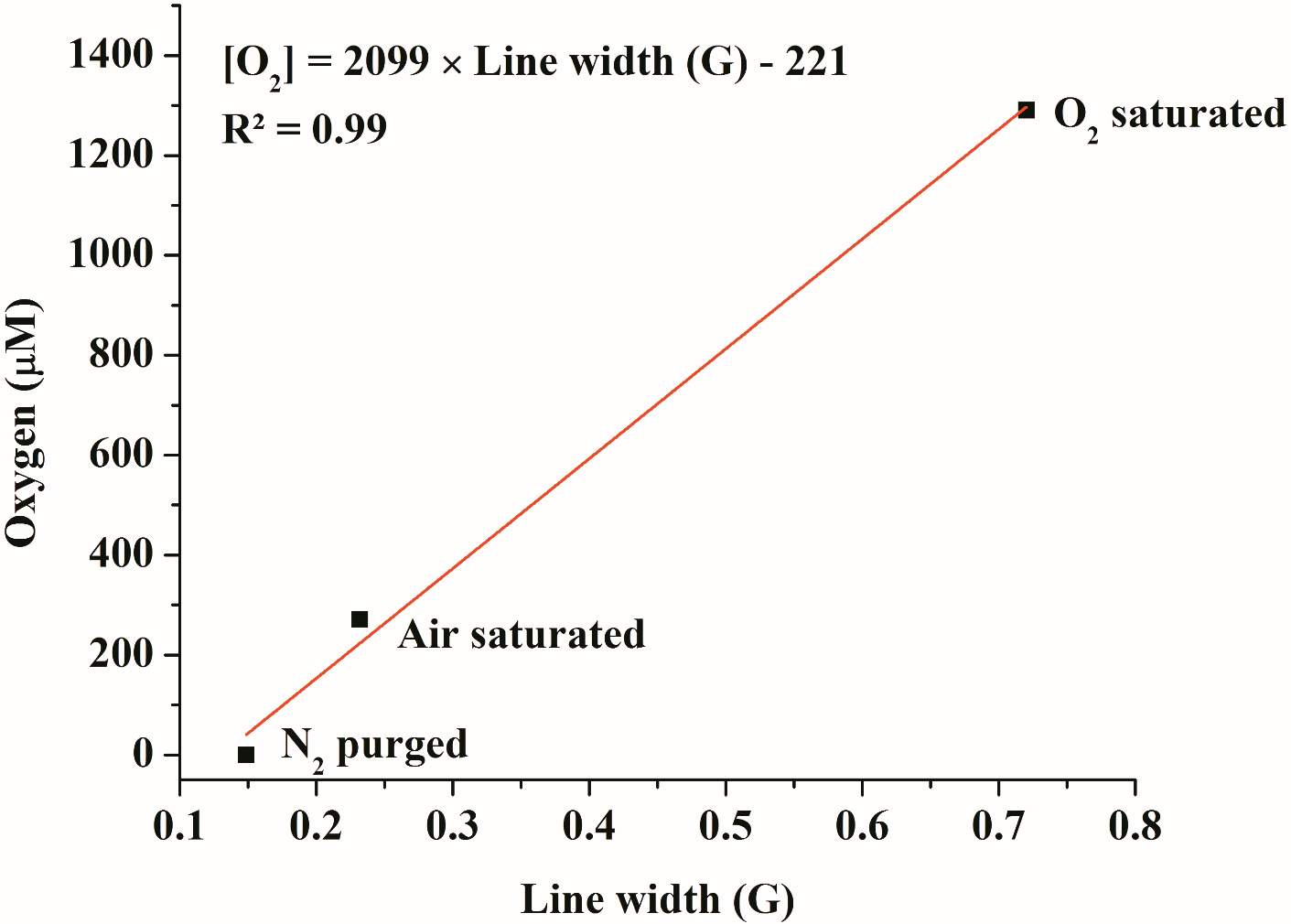
**

**Figure S1.** The linear relationship between line width of ESR spectra and O_2_ concentration in solutions. In the O_2_ saturated solution, the O_2_ concentration is 1229 μM. While in the air saturated solution, the O_2_ concentration is 258 μM.





**Figure S2**. Transmission electron microscopy images and the corresponding size distribution histograms of (a) Au-5nm/TA, (b) Au-10nm/TA, (c) Au-15nm/TA, (d) Au-30nm/TA, (e) Au-60nm/TA, (f) Au-5nm/Citrate, and (g) Au-5nm/PVP.





**Figure S3**. Rarefaction curves calculated for each sample.


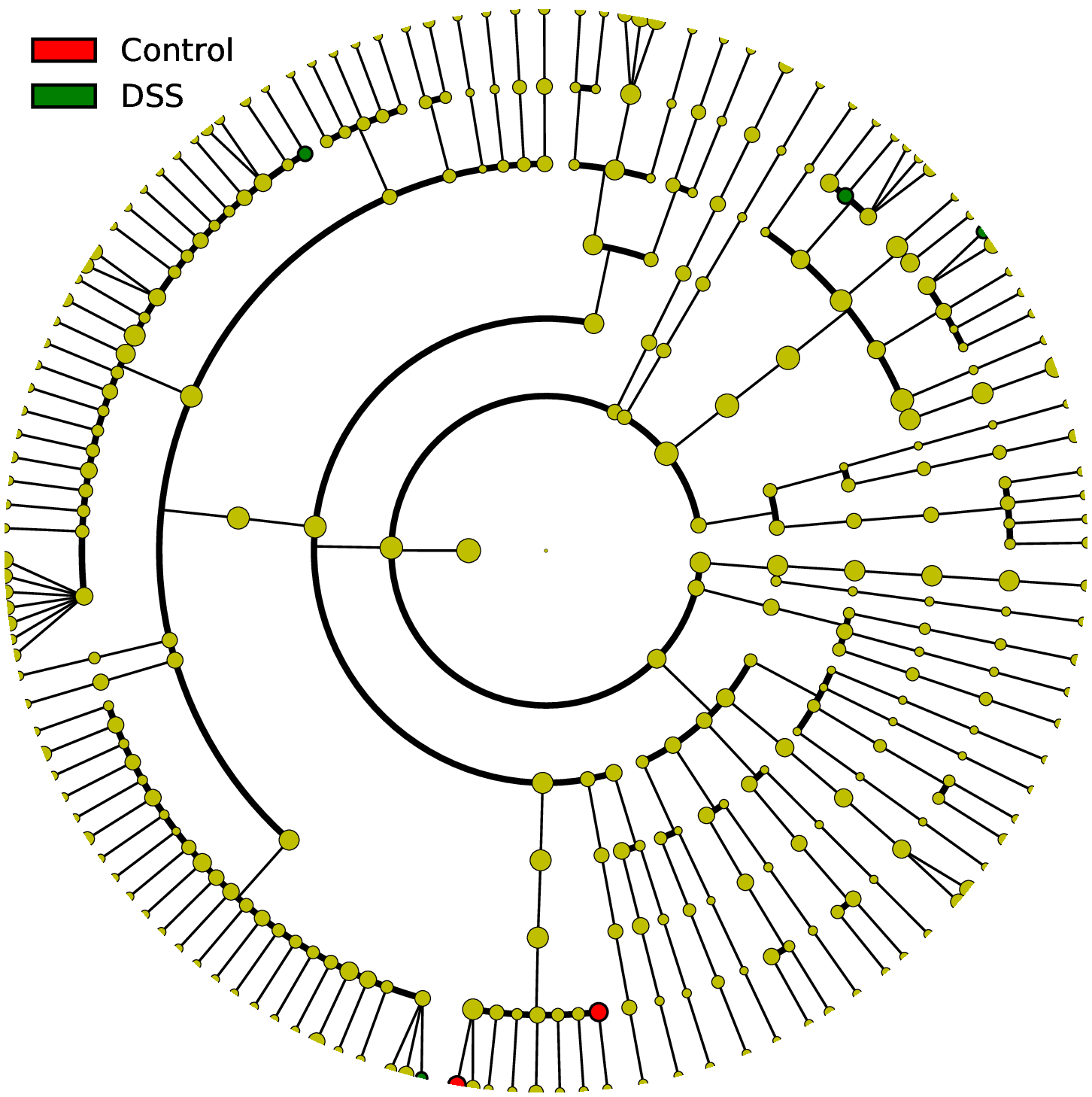


**Figure S4.** Taxonomic cladogram generated from LEfSe analysis showing significant difference in microbiota profile of the groups of normal control and DSS control, red represented the enriched taxa in the control group’s microbial community and green represented the enriched taxa in the DSS group’s microbial community.
